# Antimicrobial activity and brine shrimp toxicity of extracts of *Terminalia brownii *roots and stem

**DOI:** 10.1186/1472-6882-7-9

**Published:** 2007-03-30

**Authors:** Zakaria H Mbwambo, Mainen J Moshi, Pax J Masimba, Modest C Kapingu, Ramadhani SO Nondo

**Affiliations:** 1 Institute of Traditional Medicine, Muhimbili University College of Health Sciences, P.O. Box 65001, Dar es Salaam, Tanzania

## Abstract

**Background:**

*Ternimalia brownii *Fresen (Combretaceae) is widely used in traditional medicine to treat bacterial, fungal and viral infections. There is a need to evaluate extracts of this plant in order to provide scientific proof for it's wide application in traditional medicine system.

**Methods:**

Extraction of stem bark, wood and whole roots of *T. brownii *using solvents of increasing polarity, namely, Pet ether, dichloromethane, dichloromethane: methanol (1:1), methanol and aqua, respectively, afforded dry extracts. The extracts were tested for antifungal and antibacterial activity and for brine shrimp toxicity test.

**Results:**

Extracts of the stem bark, wood and whole roots of *T. brownii *exhibited antibacterial activity against standard strains of *Staphylococcus aureus*, *Escherichia coli*, *Pseudomonas aeruginosa, Klebsiella pneumoniae*, *Salmonella typhi*, and *Bacillus anthracis *and the fungi, *Candida albicans *and *Cryptococcus neoformans*. Aqueous extracts exhibited the strongest activity against both bacteria and fungi. Extracts of the roots and stem bark exhibited relatively mild cytotoxic activity against brine shrimp larvae with LC_50 _values ranging from 113.75–4356.76 and 36.12–1458.81 μg/ml, respectively. The stem wood extracts exhibited the highest toxicity against the shrimps (LC_50 _values 2.58–14.88 μg/ml), while that of cyclophosphamide, a standard anticancer drug, was 16.33 (10.60–25.15) μg/ml.

**Conclusion:**

These test results support traditional medicinal use of, especially, aqueous extracts for the treatment of conditions such as diarrhea, and gonorrhea. The brine shrimp results depict the general trend among plants of the genus *Terminalia*, which are known to contain cytotoxic compounds such as hydrolysable tannins. These results warrant follow-up through bioassay-directed isolation of the active principles.

## Background

*Terminalia brownii *Fries (Combretaceae) is found in many parts of Africa and it has different uses. It is found in the Democratic Republic of Congo, Ethiopia, Kenya, Tanzania [1,2]. In Tanzania the plant grows in Morogoro, Coast Region, Tanga and Arusha. It has different vernacular names in different places such as kuuku, muvuku (Kamba, Kenya), koloswa (northern region, Kenya), weba (Ethiopia), lbukoi (Samburu, Kenya), orbukoi (Maasai, Tanzania), and mbarao or mwalambe, in Kiswahili. The leaves are used by traditional healers in Tanzania to treat diarrhoea and stomach ache, gastric ulcers, colic, and heartburn [2,3]. In the Democratic Republic of Congo barks from the stems, branches, and trunks are used to treat urogenital infections, urethral pain, endometritis, cystitis, leucorrhoea, syphilis, and gonorrhoea [4]. It is also used by traditional healers in Kenya to treat malaria [5]. The decoction of the stem bark, trunk and branches is taken orally to treat dysmenorrhoea, nervosity, hysteria, epilepsy, beriberi, dyspepsia, stomachache, gastric ulcers, and colitis [2,6]. Stem barks are chewed to treat cough and as emetic, infusion of barks and leaves are mixed with meat to treat hepatitis [7]. Traditional healers in Ethiopia use the stem and barks to treat jaundice, hepatitis, liver cirrhosis, and yellow fever [8-10].

## Methods

### Materials

Petroleum ether, dichloromethane, and methanol were purchased from Fisher Scientific, UK, Ltd (Bishop Meadow Road, Loughborough, Leicestershire, LE 11 5RG, UK). Saboraud's dextrose agar (SDA) and Mueller Hinton agar were purchased from Oxoid Ltd (Basingstoke, Hampshire, England), while dimethylsulfoxide (DMSO) was purchased from Sigma (Poole, Dorset, England). Brine shrimp eggs were bought from Dohse Acquaristic, Bonn (Aus Dem Hause Dohse Acquaristik), Germany. Cyclophosphamide, Gentamicin susceptibility test discs (10 μg) and Clotrimazole (20 μg), were purchased from Oxoid Ltd (Basingstoke, Hampshire, England). Sea salt was prepared locally by evaporating water collected from the Indian Ocean, along the Dar es Salaam Coast.

### Collection of Plant material

*Ternimalia brownii *Fresen (Combretaceae) roots and stem were collected in Mombo, Tanga Region, Tanzania. The plant was identified by Haji, Selemani of Department of Botany, University Dar es salaam, and the voucher specimen no. RKR 222 is kept in the Herbarium of the Institute of Traditional Medicine, Muhimbili University College of Health Sciences.

### Preparation and extraction of plant material

Powdered air-dried stem bark of *T. brownii *(500 g) was defatted using petroleum ether by maceration, overnight, to afford oily extract (0.69 g). Then the material was subjected to sequential extraction using solvents of increasing polarity to afford dry extracts of dichloromethane (1.54 g), 1:1 dicloromethane:methanol (3.96), methanol (33.27 g) and water (18.05 g). Powdered air-dried stem wood (500 g) and roots (500 g) were similarly extracted leading to extracts of petroleum ether (2.0; 2.0 g), dichloromethane (3.5; 2.0 g), 1:1 dichloromethane:methanol (8; 14.0 g), methanol (34; 17.0 g), and water (3.0; 6.5 g), respectively.

### Antimicrobial tests

Antibacterial and antifungal activities were tested by the disc-diffusion method [11]. Eight standard bacteria, *Staphylococcus aureus *(NCTC 6571), *Escherichia coli *(NCTC 10418), *Pseudomonas aeruginosa *(NCTC 10662), *Klebsiella pneumoniae *(NCTC 9633), *Salmonella typhi *(NCTC 8385), *Bacillus anthracis *(NCTC 10073), *Bacillus cereus *(NCTC 7464), and *Proteus mirabilis *(NCTC 10975) and the fungi, *Candida albicans *(Strain HG 392), and local strains of *Cryptococcus neoformans *extracted from hospitalized patient were used. Filter paper discs (Whatman No. 1; 5 mm diameter) were impregnated with crude extracts (5 mg/disc) or standard drugs (10 μg/disc gentamicin; for bacteria) and (20 μg/disc clotrimazole; for fungi). The discs were overlayed on tryptone soya agar plates (for bacteria) and Saborauld's dextrose agar plates (for fungi) and incubated at 37°C, for 24 h. The discs were tested in triplicate, including one with a solvent blank and 3 for the standard drugs. Inhibition zones were calculated as the difference between disc diameter (5 mm) and the diameters of inhibition [12]. The mean inhibition zones were used to calculate the activity index. Activity index (AI) was calculated as the mean inhibition zone for test sample divided by the mean inhibition zone for the standard drug [11].

### Brine shrimp lethality test

The brine shrimp lethality test (BST) was used to predict the presence, in the extracts, of cytotoxic activity [13]. Assay procedures and analysis of results was done as reported earlier [14]. Cyclophosphamide was used a standard anticancer test drug.

## Results

### Anti-microbial activity

With the exception of Petroleum ether extracts, all the other extracts of stem bark were active against one or more of the tested organisms (Tables 1 and 2). The best activity of the stem bark extracts was exhibited by methanolic extract against *S. aureus *and *B. anthracis*; aqueous extract against *S. aureus*, *B. anthracis*, *K. pneumoniae *and both *C. albicans *and *C. neoformans*. Similarly, in the case of stem wood extracts, the Petroleum ether extract was inactive against all tested organisms whereas all other extracts exhibited activity against a number of tested organisms with the best activity being recorded for against *B. anthracis*, *B. cereus, S. aureus*, *C. albicans *and *C. neofaormans*. All the root extracts tested (Table 3) exhibited activity against *S. aureus, P. aeruginosa, B. anthracis*, and *C. albicans*, but none of them showed activity against *S. typhi*. The root aqueous extract exhibited activity against *S. aureus, P. aeruginosa, B. anthracis, B. cereus, K. pneumoniae, C. albicans *and *C. neoformans*. It was, however, inactive against *P. mirabilis, S. typhi*, and *E. coli*.

**Table 1:** Antimicrobial activity of *T. brownii *stem bark extracts

**Extracts/drugs**		**Organisms tested**									
		
		*S. aureus*	*P. mirabilis*	*S. typhi*	*E. coli*	*P. aeruginosa*	*B. anthracis*	*B. cereus*	*K. pneumoniae*	*C. albicans*	*C. neoformans*
PE	IZ	-	-	-	-	-	-	-	-	-	-
	AI	-	-	-	-	-	-	-	-	-	-
DM	IZ	-	3.0 ± 1.0	-	-	5.0 ± 1.0	4.3 ± 0.6	-	-	-	-
	AI	-	0.25	-	-	0.37	0.24	-	-	-	-
DM:M (1:1)	IZ	7.3 ± 1.1	7.3 ± 0.6	-	6.0 ± 0.0	-	4.7 ± 0.6	-	-	5.3 ± 0.6	4.7 ± 0.6
	AI	0.52	0.61		0.50	-	0.26	-	-	0.36	0.33
M	IZ	10.3 ± 0.6	-	-	-	-	13.3 ± 0.6	7.3 ± 1.1	7.3 ± 1.1	10.0 ± 0.0	6.5 ± 0.7
	AI	0.73	-	-	-	-	0.74	0.52	0.55	0.68	0.45
Aqua	IZ	11.3 ± 1.1	-	-	-	8.7 ± 0.6	13.0 ± 1.7	7.3 ± 1.1	9.3 ± 0.6	10.7 ± 0.6	11.7 ± 0.6
	AI	0.81	-	-	-	0.65	0.72	0.52	0.70	0.73	0.82
Gent	IZ	14.0 ± 0.0	12.0 ± 2.0	13.7 ± 1.1	12.0 ± 1.0	13.3 ± 1.1	18.0 ± 1.0	14.0 ± 1.0	13.3 ± 2.1	-	-
	AI	1.00	1.00	1.00	1.00	1.00	1.00	1.00	1.00	-	-
Clotr	IZ	-	-	-	-	-	-	-	-	14.7 ± 0.6	14.3 ± 0.6
	AI	-	-	-	-	-	-	-	-	1.00	1.00

The results are presented as inhibition zone diameters in mm. Each value represents mean ± SD (n = 3). Key: PE = petroleum ether extract; DM = dicloromethane extract; 1:1 D:M = 1:1 dicloromethane:methanol extract; M = methanol extract; Aqua = aqueous extract; Gent = gentamicin; Clotr = Clotrimazole.

**Table 2:** Antimicrobial activity of *T. brownii *stem wood extracts

**Extracts/drugs**		**Organisms tested**									
		
		*S. aureus*	*P. mirabilis*	*S. typhi*	*E. coli*	*P. aeruginosa*	*B. anthracis*	*B. cereus*	*K. pneumoniae*	*C. albicans*	*C. neoformans*
PE	IZ	-	-	-	-	-	-	-	-	-	-
	AI	-	-	-	-	-	-	-	-	-	-
DM	IZ	-	-	3.7 ± 1.5	7.3 ± 0.6	4.3 ± 0.6	3.3 ± 0.6	-	-	3.0 ± 1.0	-
	AI	-	-	0.34	0.46	0.29	0.21	-	-	0.19	-
DM:M (1:1)	IZ	12.0 ± 0.0	-	-	8.3 ± 0.6	-	14.7 ± 0.6	8.0 ± 0.0	-	13.3 ± 0.6	8.3 ± 0.6
	AI	0.80	-	-	0.54	-	0.92	0.63	-	0.83	0.73
M	IZ	11.3 ± 0.6	-	-	-	-	15.5 ± 0.7	11.5 ± 0.7	-	14.3 ± 0.6	10.3 ± 0.6
	AI	0.79	-	-	-	-	0.97	0.79	-	0.89	0.88
Aqua	IZ	12.0 ± 1.0	-	-	-	9.3 ± 1.5	11.7 ± 1.5	7.0 ± 1.0	6.7 ± 2.1	12.3 ± 1.5	9.7 ± 1.1
	AI	0.84	-	-	-	0.63	0.73	0.49	0.44	0.77	0.83
Gent	IZ	14.3 ± 1.5	13.0 ± 1.0	11.3 ± 0.6	15.7 ± 0.6	14.7 ± 0.6	16.0 ± 1.0	14.3 ± 0.6	15.3 ± 1.1	-	-
	AI	1.00	1.00	1.00	1.00	1.00	1.00	1.00	1.00	-	-
Clotr	IZ	-	-	-	-	-	-	-	-	16.0 ± 1.0	11.7 ± 0.6
	AI	-	-	-	-	-	-	-	-	1.00	1.00

The results are presented as inhibition zone diameters in mm. Each value represents mean ± SD (n = 3). Key: PE = petroleum ether extract; DM = dicloromethane extract; 1:1 D:M = 1:1 dicloromethane:methanol extract; M = methanol extract; Aqua = aqueous extract; Gent = gentamicin; Clotr = Clotrimazole.

**Table 3:** Antimicrobial activity of *T. brownii *root extracts

**Extracts/drugs**		**Organisms tested**									
		
		*S. aureus*	*P. mirabilis*	*S. typhi*	*E. coli*	*P. aeruginosa*	*B. anthracis*	*B. cereus*	*K. pneumoniae*	*C. albicans*	*C. neoformns*
PE	IZ	3.0 ± 0.6	11.7 ± 1.5	-	2.5 ± 1.3	4.5 ± 0.7	5.5 ± 1.7	-	2.3 ± 0.3	2.5 ± 0.5	-
	AI	0.21	0.83	-	0.17	0.32	0.30	-	0.19	0.18	-
DM	IZ	7.0 ± 2.0	12.5 ± 1.7	-	5.0 ± 0.5	4.5 ± 1.1	7.5 ± 0.7	-	1.0 ± 0.6	2.0 ± 0.7	-
	AI	0.50	0.89	-	0.33	0.32	0.43	-	0.08	0.14	-
DM:M (1:1)	IZ	11.5 ± 1.1	13.0 ± 1.0	-	-	-	-	5.5 ± 0.3	-	12.0 ± 1.0	5.5 ± 0.3
	AI	0.82	0.93	-	-	-	-	0.39	-	0.86	0.37
M	IZ	12.7 ± 1.1	13.5 ± 1.7	-	-	9.0 ± 2.0	12.0 ± 2.0	4.5 ± 0.7	-	8.0 ± 1.0	7.0 ± 1.0
	AI	0.91	0.96	-	-	0.64	0.67	0.32	-	0.57	0.47
Aqua	IZ	14.0 ± 1.1	-	-	-	12.0 ± 1.1	13.0 ± 1.0	6.5 ± 0.3	6.0 ± 1.0	6.0 ± 2.0	8.5 ± 1.5
	AI	1.00	-	-	-	0.86	0.72	0.46	0.50	0.43	0.54
Gent	IZ	14.0 ± 1.5	14.0 ± 1.0	14.0 ± 1.1	15.0 ± 1.5	14.0 ± 1.0	18.0 ± 1.0	14.0 ± 1.7	12.0 ± 1.0	-	-
	AI	1.00	1.00	1.00	1.00	1.00	1.00	1.00	1.00	-	-
Clotr	IZ	-	-	-	-	-	-	-	-	14.0 ± 2.0	15.0 ± 1.0
	AI	-	-	-	-	-	-	-	-	1.00	1.00

The results are presented as inhibition zone diameters in mm. Each value represents mean ± SD (n = 3). Key: PE = petroleum ether extract; DM = dicloromethane extract; 1:1 D:M = 1:1 dicloromethane:methanol extract; M = methanol extract; Aqua = aqueous extract; Gent = gentamicin; Clotr = Clotrimazole.

### Brine shrimp lethality test

Brine shrimp results presented (Table 4) show that the root extracts were virtually non-toxic on the shrimps. They exhibited very low toxicity, giving LC_50 _values greater than 100 μg/ml. The root aqueous extract was the most toxic. On the other hand the stem wood extracts were all very toxic to brine shrimps with LC_50 _values ranging from 2.6–14.9 μg/ml. The LC_50_values for stem bark extracts ranged between 68.4–88.0 μg/ml for dichloromethane, 1:1 dichloromethane:methanol, methanol, and aqueous extracts. The petroleum ether extract (LC_50 _1458.81 μg/ml) exhibited very low toxicity.

**Table 4:** Brine shrimp lethality tests of *Terminalia brownii *extracts of root (A), stem bark (B), and stem wood (C).

Extract type		LC50 μg/ml	95% CI
PE	**A**	285.7	189.2–431.4
	**B**	1458.81	608.60–3496.77
	**C**	13.008	8.430–20.05
DM	**A**	1462.2	684.6–3123.3
	**B**	36.12	27.36–47.68
	**C**	13.525	9.48–19.29
1:1 DM:M	**A**	4356.76	1273.9–14900.12
	**B**	70.27	51.31–86.43
	**C**	2.5857	1.23–5.44
M	**A**	409.84	253.05–663.8
	**B**	88.05l	73.56–105.40
	**C**	14.88	10.41–21.26
Aqua	**A**	113.75	88.25–146.6
	**B**	68.64	55.80–84.43
	**C**	Not Tested	--
Cyclophosphamide		16.33	10.60–25.15

The results are presented as LC_50 _values (μg/ml) and 95% Confidence Intervals (CI). Key: PE = petroleum ether extract; DM = dicloromethane extract; 1:1 D:M = 1:1 dicloromethane:methanol extract; M = methanol extract; Aqua = aqueous extract;

## Discussion

*Terminalia brownii *is used in traditional medicine to treat bacterial and viral infections [2-4,7,8,10]. The claims for treatment of gastrointestinal conditions, such as diarrhea [3] are similar to previous claims on two other plants of the genus *Terminalia *[14,15]. These claims have been supported by the current bioassay results, which have shown activity against bacterial and fungal pathogens. The root extracts were particularly more active on bacteria and fungi than the stem extracts. It is worth noting that, much as the root extracts were more active against bacteria and fungi, they exhibited very low toxicity on brine shrimps. This may suggest inherent selectivity of the extracts, thus making the root preparations more suitable for treatment of bacterial and fungal infections. It is also notable that the aqueous extracts, which in most cases are the ones used by traditional healers, were active on bacteria and fungi, and slightly less toxic on brine shrimps. The stem extracts also had antimicrobial activity, but high brine shrimp toxicity. This indicates less selectivity of the stem extracts, and hence possibility of toxicity or other biological activities such as anticancer activity [13].

One of popular use of *Terminalia *species in Tanzania is for the treatment of diarrhea, particularly in HIV patients [14,15]. The leaves of this plant are used by traditional healers in Tanzania to treat diarrhoea, stomach ache, gastric ulcers, colic, and heartburn [2,3]. Since almost all the extracts that were tested showed activity against either *Candida albicans *or *Cryptococcus neoformans*, or both, it may be speculated here that the extracts would be useful for treatment of diarrhoea caused by gastrointestinal *Candida *infection, the most frequently encountered fungal infection in HIV patients.

## Conclusion

Both organic and aqueous extracts of the stem and roots of *Terminalia brownii *have indicated varied levels of antibacterial and antifungal activity. Some of these extracts also exhibited cytotoxic activity on brine shrimps, comparable to that of a standard anticancer drug, cyclophosphamide. The traditional use of *Terminalia brownii *extracts to treat diarrhoea, cut wounds, gonnorrhea, and other infections has been supported by laboratory results from this study, suggesting a need to isolate and evaluate active constituents responsible for the exhibited biological activities.

## Authors' contributions

ZHM was responsible for study concept, designing and coordinating the research and writing the manuscript.

MJM was involved in methodological development, designing the study, data acquisition and analysis and revising the manuscript.

PJ M was involved in methodological development, data acquisition and revising the manuscript.

MCK was involved in coordinating the study and revising the manuscript.

RSON was involved in methodological development, data acquisition and revising the manuscript.

All authors read and approved the final manuscript.

## Pre-publication history

The pre-publication history for this paper can be accessed here:


